# An Intra-Abdominal Pseudocyst around a Ventriculoperitoneal Shunt due to *Streptococcus* Infection 7 Years after Shunt Surgery

**DOI:** 10.1155/2014/898510

**Published:** 2014-01-05

**Authors:** Arata Tomiyama, Jun-ichi Harashina, Hitoshi Kimura, Keisuke Ito, Yoshihiko Honda, Hiroyuki Yanai, Satoshi Iwabuchi

**Affiliations:** ^1^2nd Department of Neurosurgery, Toho University Ohashi Medical Center, 2-17-6 Ohashi, Meguro-ku, Tokyo 153-8515, Japan; ^2^Department of Neurosurgery, Maruyama Memorial General Hospital, 2-10-5 Hon-cho, Iwatsuki-ku, Saitama-shi, Saitama 339-8521, Japan

## Abstract

In 1999, a 50-year-old woman underwent ventriculoperitoneal (VP) shunt surgery for hydrocephalus after subarachnoid hemorrhage. She was hospitalized for fever and recurrent systemic seizures in November 2006. Head computed tomography (CT) showed only old changes. The seizures and fever were controlled by medicinal therapy. However, in December, her consciousness level suddenly decreased, and she showed progressive lower abdominal distension. Head CT showed marked ventriculomegaly, and abdominal CT showed a giant cystic mass at the shunt-tube tip in the lower abdominal cavity. Because thick pus was aspirated from the intra-abdominal mass, we diagnosed the patient with acute obstructive hydrocephalus due to an infected abdominal pseudocyst. Laparotomy and direct cyst drainage were performed, and antibiotic therapy against *Streptococcus,* the causative pathogen, was administered. The VP shunt tube was replaced. The postoperative course was uneventful, and postoperative CT showed hydrocephalus improvement and no pseudocyst recurrence. Abdominal pseudocysts, which are rare after VP shunt surgeries, usually occur after the subacute postoperative course in younger cerebral hemorrhagic cases. Our case was quite rare because the cyst developed in the chronic phase in an older patient and was caused by streptococcal infection. The cyst components should be examined before cyst drainage when choosing surgical strategies.

## 1. Introduction

Complications after ventriculoperitoneal (VP) shunt operations are observed relatively often. However, among these complications, intraperitoneal pseudocysts (IPP) are rare. We describe a case of an IPP that occurred 7 years after a VP shunt operation in a patient who was infected with pseudomonas and who presented with acute hydrocephalus due to shunt malfunction.

## 2. Case Presentation

A 50-year-old female was transferred to our hospital in November 2006 because of recurrent attacks of epilepsy and fever. In 1999, she had undergone an aneurysm clipping surgery after subarachnoid hemorrhage (SAH) in our institute and had also had a VP shunt operation against SAH-induced hydrocephalus after a month from initial clipping. After that, she received long-term rehabilitation after being transferred to another hospital.

**Figure 1:**
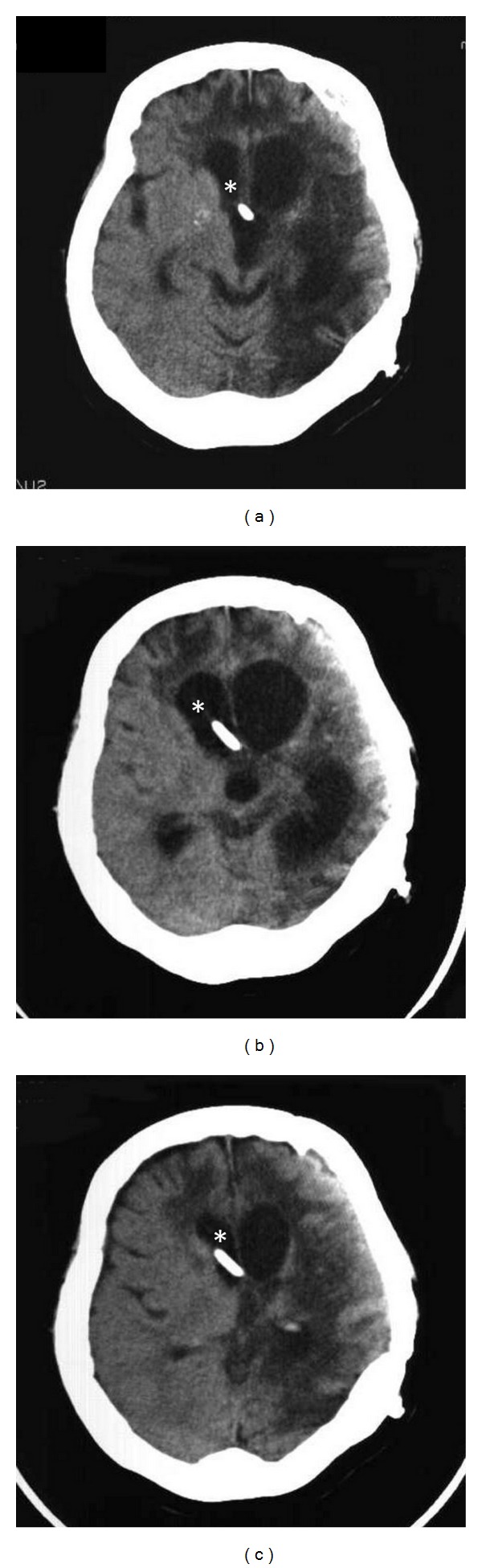
Head computer tomographic (CT) scan image that was performed at the time of admission (a) indicating previous destructive changes in the brain due to damage from the prior subarachnoid hemorrhage and vasospasms with mild hydrocephalus. Head CT scan image showing acute deterioration (b) indicating progression of the hydrocephalus. Head CT scan image after the ventriculoperitoneal (VP) shunt revision (c) showing improvement of the hydrocephalus. In each head CT scan image ((a), (b), or (c)), the VP shunt tube can be observed (∗).

Upon admission to our hospital, the patient's consciousness level was impaired (Glasgow Coma Scale, E4V2 M5), and hemiplegia of the right side due to a cerebral infarction that was triggered by vasospasms after the prior SAH was observed. Frequent clonic epilepsy attacks occurred daily on the left side of the body, including the face. A hematological examination revealed a mild upregulation of inflammation markers (white blood cells, 12,000 cells/*μ*L; C-reactive protein levels: 5.2 mg/dL). Head computed tomographic (CT) scans that were performed upon admission showed mild hydrocephalus and ischemic lesions that were due to damage from the vasospasms that occurred after the SAH (Figure 1(a)). After admission, the epilepsy was relieved by the administration of anticonvulsants. However, the fever, which was around 37°C and 38°C, was sustained. The focus of the fever could not be detected despite systematic examination.

**Figure 2:**
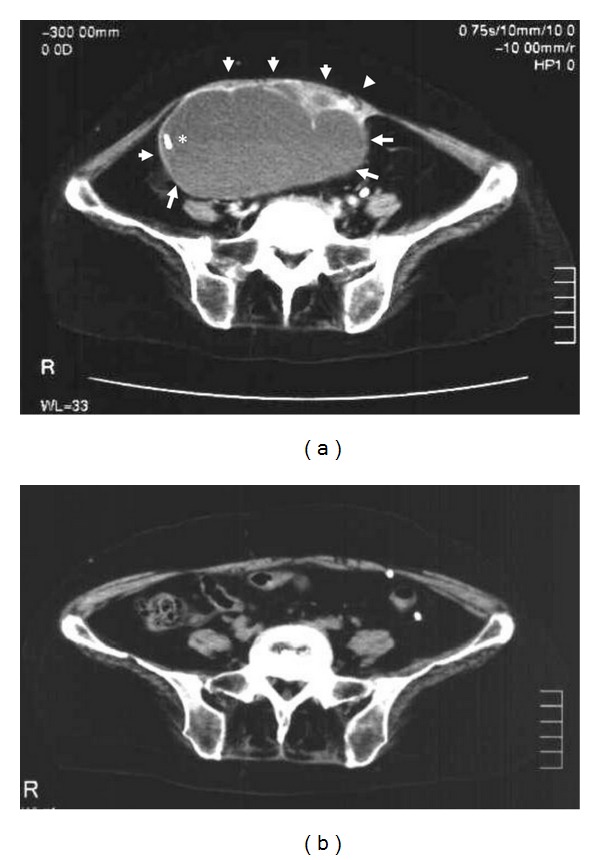
Preoperative abdominal CT scan image with contrast medium (a) showing a huge multilocular cyst-like enhanced mass (arrowheads) in the peritoneal space around the distal tip of the VP shunt tube (**∗**). An abdominal CT image that was performed 2 months after the laparotomy (b) indicating no recurrence of the pseudocyst.

In December, the patient exhibited a sudden deterioration in consciousness (Glasgow Coma Scale, E2V1 M4) and abdominal distention. Head CT scans revealed worsening of the hydrocephalus (Figure 1(b)), and an abdominal CT scan study with contrast medium demonstrated a large intra-abdominal cyst (>10 cm) that was enhanced in a multilocular cystic pattern and that was located at the abdominal distal tip of the VP shunt tube (Figure 2(a)). In addition, echogram-guided aspiration of the components of the cyst revealed massive amounts of pus. Therefore, we diagnosed the patient with the rapid development of hydrocephalus due to malfunction of the VP shunt tube that was caused by the formation and infection of the IPP at the abdominal tip of the VP shunt tube. Extracorporeal drainage of the cyst was done while the patient was under general anesthesia in January 2007. At the same time, the abdominal side of the VP shunt tube, including the shunt valve, was removed, and the remaining end of the tube of the VP shunt system that was on the head side was reputed as external drainage. At that time, no infection of the cerebrospinal fluid was detected. However, cultures of the abdominal pus revealed *Streptococcus pneumoniae* infection. Therefore, *Streptococcus*-sensitive vancomycin (0.5 g × 2/day) was administered continuously. A week after the first operation, the patient's systemic status was stabilized, and the pus discharge from the cyst drainage stopped. Thus, the VP shunt tube was replaced, and the cyst drainage was removed while the patient was under general anesthesia. The postoperative course was uneventful, and a head CT scan that was performed a month after the surgery revealed improvement of the hydrocephalus (Figure 1(c)). A postoperative abdominal CT scan that was performed two months after the surgery demonstrated no recurrence of the IPP (Figure 2(b)).

## 3. Discussion

The frequency of complications after VP shunt surgery has been reported to be around 25%, and, among these complications, the incidence of IPP is low with rates that have been reported to range from 0.33% to 6.8% since it was first reported in 1954 [1–3]. The exact pathogenesis of IPPs after VP shunts is still unknown. However, past reports have documented that the pathogenesis of IPPs involve local inflammation around the VP shunt tube in the abdomen [2, 4, 5]. In addition, the involvement of other factors, including intra-abdominal tumors, a history of abdominal operations, abnormalities of the ascites, such as disturbances in absorption or abnormalities of the constituents, and silicon allergies, has also been reported [4–6].

Our adult case exhibited onset seven years after the VP shunt surgery. This case is considered a rare case of IPP because IPPs that are associated with VP shunts usually occur 3 weeks to 5 years after VP shunt surgeries in younger patients [7, 8]. Thus, it is important to consider an IPP as a candidate cause of acute malfunction of a VP shunt system when more than 5 years have passed since the operation.

IPPs after VP shunts are usually diagnosed with abdominal echograms or abdominal CT scans [2, 8]. However, it has also been reported that distinguishing IPPs from lymphoid cysts, abscesses, cerebrospinal fluid collection, seromas, or urinomas is sometimes difficult with imaging, and echogram-guided aspirations of the cyst components, which were useful for our case, are a more reliable method for the diagnosis of such cases [9].

The cyst components of IPPs that are associated with VP shunts are often infected, and the pathogens have usually been reported as dermal resident flora, such as *Staphylococcus aureus* or *Staphylococcus epidermidis *[2, 10]. Unlike typical cases, the pathogen in our case was *Streptococcus pneumoniae, *and this is the first reported case of this type of infection, as far as we know. Thus, these findings suggested that not only dermal resident flora but also *Streptococcus pneumoniae* are important potential candidates as pathogens of IPP infection.

Cyst drainage or cyst opening by laparoscopic or direct surgery is usually chosen as the therapeutic strategy for the treatment of IPPs after VP shunt surgery. In particular in cases with uninfected cysts, only the removal of the tube tip from the cyst has been considered an effective method in recent years [5, 6, 11]. However, it has also been reported that not only tube removal from the cyst but also direct cyst drainage or removal is necessary when cyst infection has been confirmed at a stage of preoperative diagnosis, as in our case [5, 6]. Therefore, the preoperative diagnosis of cyst infections by methods, such as cyst puncture, is critical for determining the most appropriate surgical procedure for the treatment of IPPs that are associated with VP shunts.
